# Anti-inflammatory and Apoptotic Effects of Levisticum Officinale Koch Extracts on HT 29 and Caco-2 Human Colorectal Carcinoma Cell Lines

**DOI:** 10.31661/gmj.v13i.3341

**Published:** 2024-04-07

**Authors:** Marzieh Lotfian Sargazi, Zahra Miri Karam, Ali Shahraki, Mahboobeh Raeiszadeh, Mohammad Javad Rezazadeh Khabaz, Abolfazl Yari

**Affiliations:** ^1^ Physiology Research Center, Institute of Neuropharmacology, Kerman University of Medical Sciences, Kerman, Iran; ^2^ Physiology Research Center, Institute of Basic and Clinical Physiology Sciences, Kerman University of Medical Sciences, Kerman, Iran; ^3^ Department of Biology, Faculty of Science, University of Sistan and Baluchestan, Zahedan, Iran; ^4^ Herbal and traditional medicines research center, Kerman University of Medical Sciences, kerman, Iran; ^5^ Department of Medical Genetics, Faculty of Medicine, Shahid Sadoughi University of Medical Sciences, Yazd, Iran; ^6^ Cellular and Molecular Research Center, Birjand University of Medical Sciences, Birjand, Iran

**Keywords:** Colorectal Cancer, Levisticum Officinale Koch, Lovage, Apoptosis, Anti-cancer, Anti-inflammation

## Abstract

Background: Colorectal cancer is among the deadliest cancers in the world. Due to the occurrence of side effects related to current standard therapy, researchers are seeking better alternative treatments. For many years, herbs have been a promising source for discovering therapeutic compounds. Therefore, the primary objective of this research was to examine the distinctive apoptotic and anti-inflammatory properties exhibited by Levisticum officinale Koch (lovage) on HT-29 and Caco-2 cell lines. Materials and Methods: The maceration method was used to prepare different extracts (ethanol, dichloromethane, petroleum, and residues) from the plant. These extracts were then tested on two colon cancer cell lines - HT-29 and Caco-2 - using the MTT assay to determine the half-maximal inhibitory concentration (IC50) values. In addition, we evaluated the expression levels of several inflammatory genes (IKKb, IKKa, and REIB) using real-time PCR. We also assessed Cox-2 protein expression using western blot analysis. The western blot was also used to analyze apoptosis-related proteins, including Caspase-3, BAX, and Bcl-2. Results: The dichloromethane extract of Levisticum officin (DELO) exhibited a high cytotoxic effect on Caco-2 and HT-29 cell lines, with IC50 values of 106.0±2 μg/mL in HT-29 cells and 175.3±4 μg/mL in Caco-2 cells after 72 hours. None of the lovage extracts showed a significant cytotoxic effect on non-cancerous cells (3T3 cell line). Furthermore, the group treated with DELO showed a lower expression level of inflammatory genes and COX-2 protein compared to the control group. Notably, treatment with DELO resulted in an increase in Caspase-3 protein and BAX/Bcl-2 ratio in both HT-29 and Caco-2 cells. Conclusion: According to this study, DELO has the potential to act as an anti-inflammatory and anti-cancer agent. Further research on the compounds present in DELO and their effect on various signaling pathways could help in the development of new drugs for diseases where inflammation or cells escape from apoptosis play a crucial role.

## Introduction

Colorectal cancer (CRC) ranks among the prevalent malignancies globally, accounting for more than 8% of all fatalities each year worldwide [1]. In recent years, the incidence and death rate of this disease have significantly increased in the Iranian population [2]. Studies have shown that medicinal herbs have the potential to inhibit vital processes in cancerous cells, such as apoptotic pathways, and may be effective in preventing various forms of cancer. While surgery is the primary method used to treat CRC, many patients in the advanced stages of the disease may also benefit from chemotherapy as an adjuvant treatment [3][4][5]. Before the advent of modern pharmacy, plants were the primary source of medication. Today, herbal remedies are often viewed as less invasive and safer forms of therapy [6]. The medicinal benefits of these remedies are derived from their secondary metabolites, which play no role in plant growth or reproduction [7]. In fact, a large number of drugs currently used to treat cancer are of herbal origin [8]. These substances have the ability to specifically target metabolic, apoptotic, oxidative, and inflammatory pathways in cancer cells [9].

The Apiaceae (Umbelliferae) plant lovage, Levisticum officinale Koch, plays crucial roles in the pharmacological, chemical, and medicinal fields. This plant is mostly found in certain areas of Iran and Afghanistan such as the heights of the Hezar Mountains (Kerman, Iran) [10][11]. Its roots and leaves have been utilized in cosmetic industries as well as medicine as a diuretic, anti-culinary, anti-spasmodic, and anti-rheumatism [12]. Additionally, it has antiseptic and antibacterial functions and also is used to relieve migraine headaches [13]. It has been demonstrated that the lovage formulations have anti-inflammatory, antioxidant, and apoptotic effects on cancerous cells [14][15][16][17]. According to a study, it has been observed that lovage essential oil actively influences the expression of genes related to apoptosis and cancers. Additionally, it also plays a significant role in the regulation of ERK5 and p53 signaling pathways [14].

The whole body of lovage is aromatic and has a variety of therapeutic benefits. The main components of lovage are Phthalides and terpenes [18]. Phthalides are found in the essential oils of Levisticum officinale W.D.J. Koch and have antibacterial, antifungal, anti-inflammatory, and antioxidant properties. Furthermore, scientific evidence has demonstrated that Z-ligustilide, a specific type of monomeric phthalide, exhibits remarkable anti-tumor, anti-inflammatory, and antioxidant properties [19].

Terpenes, particularly monoterpenes, are commonly found in Levisticum officinale W.D.J. Koch. Studies have shown that α-pinene, β-pinene, myrcene, and limonene have anti-cancer and apoptotic effects [20][21][22][23]. Flavonoids are another important chemical composition in this plant, with quercetin, chlorogenic acid, caffeic acid, and luteolin being the most prominent [24]. Multiple research studies have extensively revealed the anti-cancer properties of quercetin against a wide range of cancer cell types, encompassing breast, colon, prostate, ovary, endometrium, and lung tumors [10]. Furthermore, studies have shown that chlorogenic acid, caffeic acid, and ferulic acid extracted from lovage have strong antioxidant effects [11]. Research has shown that chlorogenic acid has anti-inflammatory properties [25] and the ability to inhibit the NF-κB pathway [26][27].

Due to the potential therapeutic benefits of Levisticum officinale Koch for a variety of diseases, we conducted this study to investigate the cytotoxic, anti-inflammatory, and apoptotic effects of plant extracts on colorectal cancer cell lines.

## Materials and Methods

Plant Materials, Chemicals and Reagent Kits

In the spring of 2020, fresh aerial parts of lovage were gathered from Hezar mountain in Kerman, Iran. Cell culture reagents, including Dulbecco’s Modified Eagle’s Medium (DMEM/F12), penicillin-streptomycin, fetal bovine serum (FBS), and Trypsin-EDTA solution, were procured from GIBCO company in New York, USA. The 3-(4,5-Dimethylthiazol-2-yl)-2,5-Diphenyltetrazolium Bromide (MTT) powder, dimethyl sulfoxide (DMSO), ethanol, and TRIzol solutions were purchased from Merck KGaA in Darmstadt, Germany. Polyclonal antibodies targeting β-actin, cleaved caspase-3, B-cell lymphoma 2 (BCL-2), BCL-2 associated X (BAX), and Cyclooxygenases-2 (COX-2) were sourced from Santa Cruz Biotechnology, Inc. in California, USA. Real-time PCR was conducted using High ROX RealQ Plus Master Mix Green (2X) from Ampliqon in Denmark. The cDNA synthesis kit was obtained from Pars Toos in Mashhad, Iran. Radio immunoprecipitation assay (RIPA) buffer and phenyl methane sulfonyl fluoride (PMSF) were provided by Roche (Applied Science, Penzberg, Germany). The remaining chemicals and reagents employed in this study were all of analytical grade quality.

Herbal Extraction

The aerial parts of lovage were subjected to authentication by a certified herbalist, and a representative sample of the specimen (voucher number: KF1470) was diligently preserved at the herbarium of the Pharmacognosy department (Kerman Medical University, Iran), ensuring its availability for future reference. The air-dried plant leaves were used for extraction. The dried leaves were ground to obtain a fine powder and the maceration process was used to extract lovage. 200 gr of the powder was mixed with 1L of 80% ethanol on a shaker (Behdad, Iran). After 24 hours, the Ethanolic Extract of Levisticum officinale (EELO) was filtered and separated three times. Afterward, EELO was condensed in a rotary evaporator (LABOROTA-4011, Heidolph, Germany) at 50 °C and finally desiccated and dried in a freeze dryer (Dena Vacuum, Iran) for 24h. Fractionation is the sequential extraction of components utilizing either extracting solvents carried out by altering the polarity of the solvents to produce an effective fraction and determine the primary components of it [28]. To prepare various fractions, a portion of the dried EELO is dissolved in 50% ethanol before being added to petroleum ether and thoroughly mixed in a decanter funnel. This produces the petroleum ether phase (PELO), which is then separated. Typically, this procedure is carried out three to five times until the petroleum ether phase turns colorless. Following the drying process in an oven at 50°C, the extract is further dried using a petroleum ether solvent. The residual extract in the funnel was then mixed with dichloromethane solvents, and the dichloromethane was separated and dried separately in an oven using the same procedure as previously. Finally, the residual extract in the oven is dried along with the solution in the funnel. Until usage, the extracted powders are kept at -20 °C.

Cell Culture

The colorectal cancer cell lines (Caco-2 and HT-29) and mouse embryonic fibroblasts cell line (3T3) were purchased from the Iranian Biological Research Center (Tehran, Iran). All cell lines were cultured in T-25 flasks (SPL, Korea) containing DMEM/F12 medium, 10% FBS and 1% 100 U/ml penicillin/100 mg/ml streptomycin solution, then incubated at 37 °C in a humid environment with 5% CO2 until confluence reached 80-90%. The cells were passaged for 2-3 times and were used for subsequent analyses.

MTT Cytotoxicity Assay

At the onset, a total of 10,000 cells were carefully seeded into individual wells of a 96-well plate and given a 24-hour incubation period for proper adherence. Following that, the cells were exposed to different concentrations of all extracts (ranging from 0 to 1000 µg/mL) for varying time intervals (24 hours, 48 hours, and 72 hours) in triplicate.

Then, 20 µl of DMEM/F12 medium containing MTT dye (5 mg/mL) was carefully introduced into each well, followed by a 4-hour incubation period. After removal of the solution from the wells, 100 µl of DMSO solution was introduced to each well. The plates were subjected to gentle shaking for a period of 2 minutes, following which the absorbance of each well was measured at a wavelength of 570 nm using an ELISA plate reader (Epoch, BioTek Instruments Inc., VT, USA).

RNA Extraction and RT-PCR

RNA extraction from both the treated and control cells was performed using the TRIzol solution according to a well-described protocol [29]. A Nano Drop 2000-c spectrophotometer (Thermo Fisher Scientific, USA) was used to assess the concentration and quality of the extracted RNA. Then, the Pars Toos cDNA synthesis kit was used to create the first strand of cDNA from the total RNA as per the manufacturer’s protocol.

The samples were incubated in a FlexCycler2 PCR thermal cycler (Analytik Jena, Germany) in the following conditions: 25 °C for 10 min, 47 °C for 60 min and a stop reaction at 85 °C for 5 min. The obtained cDNA samples were kept in a deep freezer until used. A StepOne Real-Time PCR system (Applied Biosystems, USA) was used to conduct PCR amplification and evaluate the expression of the target genes. The expression of GAPDH was used as an endogenous control. The PCR parameters were set as follows: an initial denaturation phase for one cycle at 95 °C lasting 15 minutes (hold cycle), succeeded by 45 cycles involving denaturation at 95 °C for 30 seconds and annealing/extension at 72 °C for 30 seconds each. The primer sequences are detailed in Table-1 [30].

Western Blotting

Firstly, cells were seeded into 6-well plates and then treated with different concentrations of lovage for 24 h. Whole-cell proteins were extracted with a lysis buffer and then quantified with the Bradford assay. After creating a mixture of the cells, the homogenates were combined with SDS sample buffer and rapidly exposed to a temperature of 95°C for 10 minutes. After being loaded and electrophoresed on 10% SDS-PAGE gels, the sample proteins were deposited at a rate of 50 g/well into a polyvinylidene difluoride (PVDF, 0.45 µm, Millipore# IPVH00010) membrane. The PVDF membrane was electroblotted, blocked in blocking solution (5% nonfat dry milk in TBST) for 1 h at room temperature, and then washed for 15 min. The membrane was incubated with a 1:1000 dilution of the primary antibodies (anti-Bcl-2, anti-Bax, cleaved caspase-3 (p17), anti-Cox-2, and anti-β-actin) and TBST buffer for an overnight at 4°C. Subsequently, horseradish peroxidase (HRP) was introduced and allowed to incubate at room temperature for a duration of 2 h. Following this, three washes with TBST were conducted, and the specific protein bands were detected using the Enhanced Chemiluminescence (ECL) Western blotting substrate from Thermo Fisher Scientific, Shanghai, China. The Image J software from Bio-Rad, USA, was employed for the analysis of the protein bands.

Statistical Methods

All data is provided as a Mean ± SEM (standard error of mean). To compare qualitative data, one-way analysis of variance (ANOVA) and Tukey multiple comparisons were employed with GraphPad Prism 8 software (San Diego, CA, USA) to find differences between the control and experimental groups. Data were considered to indicate a statistically significant difference when a value of P < 0.05 was achieved.

## Results

**Table:** Table1. Oligonucleotide Primers used for Quantitative RT-PCR

Gene		Primer sequence (5´→3´)	Primer length (bp)	T _m_ (°C)
RELB	Sense	TGTGGTGAGGATCTGCTTCCAG	22	64.1
	Anti-sense	TCGGCAAATCCGCAGCTCTGAT	22	62.5
IKKA	Sense	ACAGAGTTCTGCCCGGTCCCT	21	64.4
	Anti-sense	CTGCTGAAGTCGGGGGCAGC	20	61.0
IKKB	Sense	CGCCCAATGACCTGCCCCTG	20	62.2
	Anti-sense	GGCACCTTCCCGCAGACCAC	20	61.5
GAPDH	Sense	CCCTCTGGAAAGCTGTGG	18	57.2
	Anti-sense	AGTGGATGCAGGGATGATG	19	62.6

**Table:** Table2. IC50 Concentration of Extract for 3T3, Caco-2, and HT-29 Cell Lines for 24h, 48h, and 72h.

**Extrac** **t** **Cell line**	EELO	DELO	PELO	RELO	P -Value
			**24h**		
**3T3**	1175.7±3 µg/ml	849.8 ±7 µg/ml	1061.3±5 µg/ml	1213.5 ± 2 µg/ml	< 0.0001, < 0.0001, < 0.0001, < 0.01, respectively
**Caco-2**	747.3± 3 µg/ml	320.3 ±1 µg/ml	471±2 µg/ml	1177±5 µg/ml	< 0.0001, < 0.0001, < 0.0001, < 0.001, respectively
**HT29**	406.1 ± 4 µg/ml	250.1 ± 6 µg/ml	322.1± 7 µg/ml	1161.2 ± 1 µg/ml	< 0.0001, < 0.0001, < 0.0001, < 0.0001, respectively
			**48h**		
**3T3**	914.7 ± 2 µg/ml	616.6 ± 5 µg/ml	756.3 ± 1 µg/ml	1184.7± 4 µg/ml	<0.0001, <0.0001, <0.00001, <0.0001 respectively
**Caco-2**	590± 4 µg/ml	284± 2 µg/ml	361.6 ± 5 µg/ml	1133.0 ± 7 µ/ml	<0.0001, <0.0001, <0.0001, <0.0001, respectively
**HT29**	319.2± 3 µg/ml	157.3 ± 2 µ/ml	211± 4 µ/ml	1089 ± 1 µ/ml	<0.0001, <0.0001, <0.0001, <0.0001, respectively
			**72h**		
**3T3**	764.4 ± 5 µg/ml	394.6 ± 3 µg/ml	438 ± 1 µg/ml	1188.9 ± 6 µg/ml	<0.0001, <0.0001, <0.0001, <0.0001, respectively
**Caco-2**	300.5 ± 3 µg/ml	175.3 ± 4 µg/ml	238.3 ± 6 µg/ml	1121± 7 µg/ml	< 0.0001, < 0.0001, < 0.0001, < 0.0001, respectively
**HT29**	230.1 ± 6 µg/ml	106± 2 µg/ml	145.6 ± 7 µg/ml	629.9 ± 4 µg/ml	< 0.0001, < 0.0001, < 0.0001, < 0.0001, respectively

Each test was repeated for three times. The IC50 values were calculated using the GraphPad Prism. Statistical analysis was performed using one-way ANOVA.

**Figure-1:**
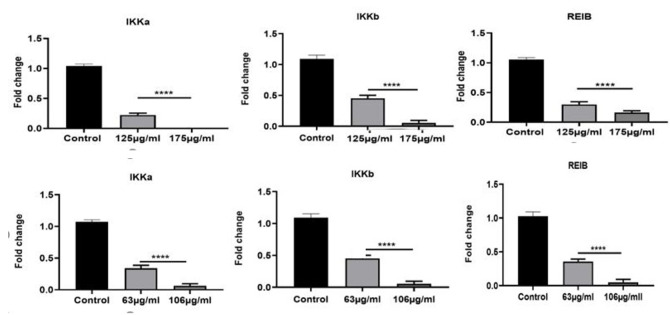
Figure 1. Relative expression of inflammatory genes including IKKa, IKKb, REIB in a) Caco-2 and b) HT29 cell lines. One-way ANOVA was employed to conduct the statistical analysis. ****P < 0.0001 was considered significantly different. Each test was repeated for three times. Graphical data denote mean ± SEM.

Dichloromethane Extract of Lovage has the Highest Cytotoxic Activity

The IC50 (50% inhibitory concentration) values of four lovage extracts including Extract DELO, EELO, Petroleum Extract of Levisticum officinale (PELO), Residual Extract of Levisticum officinale (RELO) against HT29, Caco-2, and 3T3 cell lines at 24, 48, and 72 hours are shown in Table-2. A one-way ANOVA was conducted to analyze the data statistically. At 24h, the DELO extract demonstrated the strongest cytotoxic activity against all three cell lines, with the lowest IC50 values of 849.8 ± 7 μg/ml for 3T3 cells, 320.3 ± 1 μg/ml for Caco-2 cells and 250.1 ± 6 μg/ml for HT-29 cells (P-value<0.001). This trend continued at 48h and 72h time periods as well. Among the other extracts, PELO generally showed higher cytotoxicity than EELO and RELO extracts (P-value<0.001). Importantly, the IC50 values for all extracts against the normal 3T3 cell line were significantly higher than the cancer cell lines (P-value<0.001), indicating reduced toxicity against non-cancerous cells. Based on these results, the DELO extract can be said to exhibit the most potent cytotoxic effects in a time-dependent manner against both Caco-2 and HT-29 cancer cell lines.

Effect of Extracts on the Expression of Inflammatory Genes

Analysis of qRT-PCR results with the comparative Ct (ΔΔCt) method showed inflammatory IKKa, IKKb, and REIB genes in both HT29 and Caco-2 cell lines had a significantly reduced expression in treated cells compared to non-treated cells (control) at IC50 concentrations (P-value<0.001). For HT-29 cells, a concentration-dependent significant decrease of 0.4-, and 0.1-fold in the mRNA expression of IKKa; 0.5-, and 0.1-fold in the mRNA expression of IKKb; and 0.3-, and 0.1-fold in the mRNA expression of REIB at 63, and106 μg/ml of DELO, respectively, was seen. Similarly, for the Caco-2 cells, a concentration-dependent significant decrease of 0.2, and 0.02-fold in the mRNA expression of IKKa; 0.5, and 0.01-fold in the mRNA expression of IKKb; and 0.3, and 0.1-fold in the mRNA expression of REIB at 125, and 175 μg/ml of DELO, respectively, was seen. The results were shown in Figure-1.

L. officinale Induces Apoptosis in Colorectal Cancer Cell Lntines

**Figure-2:**
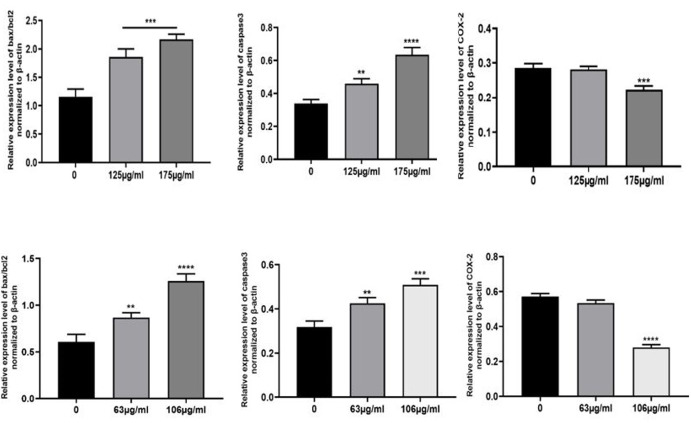
Figure 2. The protein expression level of Bax/Bcl-2 ratio, Caspase3, and COX-2 in a) Caco-2 and b) HT29 cell lines. Semi-quantitative data of protein expression of Bax, Bcl-2, and caspase-3. β-actin was included as an internal control. One-way ANOVA was employed to conduct the statistical analysis. ****P < 0.0001, ***P < 0.001, and **P < 0.01 and were considered significantly different. Each test was repeated for three times. Graphical data denote mean ± SEM.

**Figure-3:**
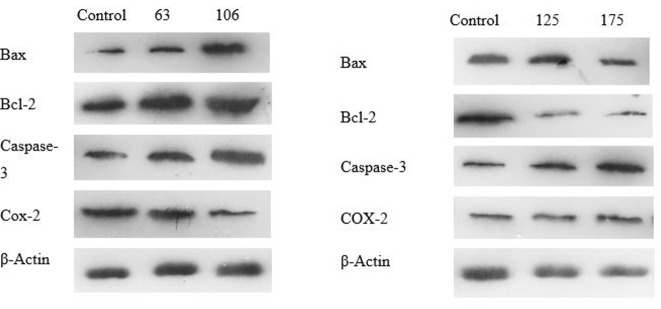
Figure 3. Effects of L. officinale on the Bax, Bcl-2, caspase-3, and COX-2 protein expression in a) HT29 and b) Caco-2 colorectal cancer cell lines. Protein expression levels of Bax, Bcl-2, caspase-3, and COX-2 were assessed through western blotting techniques.

Dysregulation apoptosis is crucial to the development and spread of cancer. The apoptosis pathway involves caspases, BAX, and Bcl-2 in a significant way. Both Caco-2 and HT-29 cells were treated with DELO and western blot analysis demonstrated that the expression level of caspase-3 was markedly decreased in these cells at both the IC50 concentration and lower dosage. Bax/Bcl-2 ratio, which is an indication of apoptosis, rose at IC50 concentration and lower dosages in both cell lines. It was also noted that Bcl-2, an anti-apoptotic protein, was expressed less and Bax, a pro-apoptotic protein, was expressed more. Additionally, we used a western blot to assess the expression of the Bax/Bcl-2 ratio. The findings presented in Figure-2 and -3 exhibit a notable elevation in the Bax/Bcl-2 ratio at concentrations of 106.0 μg/ml and 175.3 μg/ml, respectively.

## Discussion

Cancer is a complex condition that involves a delicate balance between cell growth and cell death. When pro-apoptotic genes are overexpressed or anti-apoptotic genes are under expressed, it disrupts this balance and allows dysregulated cells to evade apoptosis, leading to uncontrolled proliferation. [31]. The predominant approach in cancer therapy involves eradicating tumors through the initiation of apoptosis. Bcl-2, recognized as an anti-apoptotic member within the Bcl-2 family, serves as a key regulator of apoptosis. Functioning as a proto-oncogene, Bcl-2 hinders apoptosis by impeding the discharge of cytochrome c from the mitochondria, thereby obstructing the activation of caspases. Upon exposure to damage or stress signals, Bax monomers undergo oligomerization, resulting in the liberation of pro-apoptotic proteins and the initiation of specific caspases [32]. The ratio of Bax to Bcl-2 expression is a crucial factor in determining the fate of cells when faced with an apoptotic stimulus. A higher Bax/Bcl-2 ratio decreases the cell’s ability to resist apoptosis, resulting in increased cell death and a lower likelihood of tumor formation [33]. Caspase-3, a pivotal effector enzyme, assumes a crucial role in apoptosis by cleaving essential cellular targets responsible for chromatin condensation, DNA fragmentation, and cytoskeletal breakdown. These actions contribute to the significant morphological alterations characteristic of apoptosis [34]. Numerous studies have shown that secondary metabolites found in plant extracts possess anticancer properties by damaging DNA and inducing apoptosis in cancer cells. The advantages of natural compounds to chemotherapy drugs of unnatural origin are natural compounds which are abundant in the nature, also have lower toxicity and side effects. Therefore, it is crucial to explore herbal remedies for natural anticancer compounds in the treatment of cancer [35][36]. The primary objective of our study was to examine the cytotoxic impact of various extracts derived from lovage (petroleum, ethanolic, residual, and dichloromethane) on HT29, Caco-2 (colorectal cancer) and 3T3 cell lines (non-cancerous) using the MTT method. After determining the extract with the most cytotoxic effect on cancer cells, we used it for treatment in the subsequent steps. To investigate the anti-inflammatory effect of lovage extract, we evaluated the expression of genes involved in NF-κB (IKKa, IKKb, and REIB) and COX-2 protein in treated cancer cells compared to untreated cells (control group) by RT-PCR and western blot, respectively. Finally, we utilized the western blot technique to analyze and compare the expression levels of apoptosis-related proteins (Bax, Bcl-2, and Caspase 3) between the treatment group and the control group. The MTT assay results showed that the DELO had the highest level of cytotoxicity among all the extracts, with IC50 values of 106 µg/mL, 175 µg/mL, and 394.6 µg/mL for HT29, Caco-2, and 3T3 cell lines, respectively, after 72 hours of treatment (P-value<0.0001). According to previous studies, dichloromethane works well as a solvent for terpenes and other non-polar chemical compounds [37]. Remarkably, the lavage extracts require a significantly higher concentration to inhibit the growth of normal 3T3 fibroblast cells, compared to their effect on Caco-2 and HT-29 cells. This can be attributed to the compounds in the lovage selectively activate pathways that lead to cell death in cancer cells, while causing less harm to normal cells.

Further research is needed to understand the specific mechanisms behind this selective effect. Lovage has been shown to have cytotoxic effects on various cell lines in multiple studies. For instance, a study by Sertel et al. [14] revealed that the IC50 value of lovage essential oil was 292.6 µg/ml when tested on head and neck squamous carcinoma cells (HNSCC) using a cytotoxicity assay. ML Sargazi et al. reported hydroalcoholic extract had the IC50 value for MDA-MB-468 were 150 µg/ml and for MCF-7 200 µg/ml at 24 hours as well as significant decrease in phosphodiesterase 5 (PDE5) and intracellular cGMP in treated cells compared to untreated cells. High expression of PDE5 may play an important role in cancer. Bogucka-Kocka and colleagues [38] showed that Lovage hydroalcoholic extract had a cytotoxic effect on nine cell lines of human leukemia. Furthermore, Sargazi et al. [39] conducted a study to investigate the cytotoxic effects of lovage extract on DU-145 and PC-3 cell lines. Their results showed that the hydroalcoholic extract had cytotoxic effect on both cell lines. Additionally, Jambor et al. [40] investigated the cytotoxic effects of EELO on TM-3 mice cell lines which showed that the EELO had a concentration-dependent cytotoxic effect on TM-3 cells. While the overall evidence from these studies suggests that lovage extract possesses cytotoxic effects on cancer cell lines, it is important to consider that the specific type of extract utilized, the cell lines employed for testing, and the methodologies employed to assess cytotoxicity could potentially influence the obtained results. Several studies have shown that lovage has anti-inflammatory properties [18][24][41].

One of the most important substances found in lovage extract is ligustilide, specifically (Z)-ligustilide. The anti-inflammatory potential of ligustilides has been established through both in vitro and in vivo studies, and (Z)-ligustilide, as a crucial component, holds promise in the development of anti-inflammatory medications [12][42]. This compound works by inhibiting the NF-κB and MAPK pathways specifically in macrophage cells.

Furthermore, terpenoids have also been found to have anti-inflammatory properties and could potentially be used as medicinal treatments [43]. Studies have shown that these terpenes can inhibit NF-κB activity [44]. The transcriptional activation of NF-κB transcription factors has an important role in controlling the production of iNOS and COX-2.

In various types of cancer, particularly CRC, the NF-κB pathway is of great importance. Abnormal activation of NF-κB has been observed in more than 50% of colitis-related cancers.

This activation can contribute to tumor formation by promoting cell growth and angiogenesis, inhibiting cell death, and facilitating cell invasion and metastasis [27][44]. NF-κB dimers are associated with NF-κB protein inhibitors (I-Bs), including IKKa, IKKb, and IKK/NEMO, in the cytosol. Upon stimulation, I-Bs are phosphorylated and degraded, releasing NF-κB proteins from their inhibitory influence and allowing them to move into the nucleus [44][45]. COX-2 is induced by cytokines and growth factors and converts arachidonic acid to prostaglandin H2. Numerous studies have shown that COX-2 plays a significant role in regulating cancer-related processes such as apoptosis, invasiveness, and angiogenesis [46][47]. In fact, COX-2 mRNA expression has been found to be up to 80% higher in CRC compared to nearby non-cancerous mucosa. Furthermore, research has demonstrated that COX-2 inhibitors have the potential to prevent and reduce CRC mortality [48].

Our expression analysis indicated that the DELO decreased IKKb, IKKa, and REIB gene expression in both HT-29 and Caco-2 cells. Additionally, COX-2 protein in the HT29 and Caco-2 cell lines has dramatically lowered, according to Western blot results. Our research outcomes align with the findings of Conlon et al. [49], who found that lovage extract may lower the levels of the inflammatory cytokine generated by LPS stimulation test of THP-1 (human monocytic) cells. Another in-vitro study by Amraie et al. [50] investigated the neuroprotective effects of lovage on LPS-induced neuro inflammation.

Their findings showed that pre-treatment with lovage extract reduced hippocampal IL-6 expression levels, demonstrating the anti-inflammatory activity of this extract in LPS-induced neuro inflammation. These studies provide evidence that lovage extract may have anti-inflammatory and anti-cancer effects. It is suggested that these effects are achieved through the downregulation of genes related to inflammatory pathways.

Our Western blot analysis revealed that treatment with the DELO can induce apoptosis in the Caco-2 and HT-29 cell lines by modulating the expression of caspases, Bax, and Bcl-2 proteins. We observed a notabletablet rise in the protein level of caspase 3 and the Bax/Bcl-2 ratio within the treated groups when compared to the control group. In line with our findings, Sargazi et al. [51] reported that flow cytometry results of MDA-MB-468 and MCF-7 cells treated with hydroalcoholic extract of lovage showed a 50% increase in apoptosis rate at IC50 concentrations compared to the control group. In addition, the essential oil of lovage was found by Sertel et al. [14] to be involved in the regulation of multiple pathways, including extracellular signal-regulated kinase 5 (ERK5) signaling, integrin-linked kinase (ILK) signaling, endocytic pathways related to virus entry, and p53 signaling (a crucial tumor suppressor involved in apoptosis induction) in HNSCC.

While this study has provided valuable insights, it is essential to acknowledge its limitations. One notable constraint is the absence of in vivo experiments, which limits our ability to directly extrapolate findings to real-life biological contexts. In vivo studies would offer a more comprehensive understanding of the practical implications of our results. Additionally, the mechanisms of action underlying the observed effects remain incompletely understood, highlighting the need for further investigation in this area. Future research endeavors should delve into elucidating these mechanisms to enhance the robustness and applicability of our findings. Recognizing these limitations not only underscores the scope of our current work but also paves the way for more refined studies that can address these gaps in knowledge.

## Conclusion

In summary, our research revealed that the DELO demonstrated the highest toxicity in both HT-29 and Caco-2 cells. Additionally, we observed that the plant has anti-inflammatory properties by its impact on the expression of genes associated with the NF-κB pathway and COX-2 protein, as well as DELO has apoptotic effect. These findings, in conjunction with previous studies, suggest that lovage has potential as a treatment for cancer, Furthermore, it shows promise in the treatment of inflammation-related diseases.

## Acknowledgment

We are grateful to the study participants. This study was supported by the research grant of Kerman University of Medical Sciences, Kerman, Iran (project no. 99000878).

## Conflict of Interest

None.

## References

[R1] Bylaitė E, Venskutonis RP, Roozen JP (1998). Influence of harvesting time on the composition of volatile
components in
different anatomical parts of lovage (Levisticum officinale Koch.). J Agric Food Chem.

[R2] Farhood B, Geraily G, Alizadeh A (2018). Incidence and mortality of various cancers in Iran and compare to
other
countries: a review article. Iran J Public Health.

[R3] Yari A, Afzali A, Aalipour M, Nakheai M, Zahedi MJ (2020). KRAS and BRAF mutations in Iranian colorectal cancer patients: A
systematic review and meta-analysis. Casp J Intern Med.

[R4] Yari A, Samoudi A, Afzali A, et al (2021). Mutation status and prognostic value of KRAS and BRAF in
southeast
Iranian colorectal cancer patients: first report from southeast of Iran. J Gastrointest Cancer.

[R5] Yari A, Meybodi SME, Karam ZM, et al (2021). Association of MTHFR 677C T and 1298A C genetic polymorphisms
with
colorectal cancer: genotype and haplotype analysis in a Southeast Iranian
population. Gene Reports.

[R6] Pan SY, Litscher G, Gao SH, et al (2014). Historical perspective of traditional indigenous medical
practices: the
current renaissance and conservation of herbal resources. Evidence-based Complement Altern Med.

[R7] Pagare S, Bhatia M, Tripathi N, Pagare S, Bansal YK (2015). Secondary metabolites of plants and their role: Overview. Curr Trends Biotechnol Pharm.

[R8] Majolo F, Delwing LK, Marmitt DJ, Bustamante-Filho IC, Goettert MI (2019). Medicinal plants and bioactive natural compounds for cancer
treatment:
Important advances for drug discovery. Phytochem Lett.

[R9] Yadav VR, Prasad S, Sung B, Kannappan R, Aggarwal BB (2010). Targeting inflammatory pathways by triterpenoids for prevention
and
treatment of cancer. Toxins (Basel).

[R10] Baghel SS, Shrivastava N, Baghel RS, Agrawal P, Rajput S (2012). A review of quercetin: antioxidant and anticancer properties. World J Pharm Pharm Sci.

[R11] Ghaedi N, Pouraboli I, Askari N (2020). Antidiabetic properties of hydroalcoholic leaf and stem extract
of
Levisticum officinale: an implication for α-amylase inhibitory activity of
extract
ingredients through molecular docking. Iran J Pharm Res IJPR.

[R12] Segebrecht S, Schilcher H (1989). Ligustilide: guiding component for preparations of Levisticum
officinale
roots. Planta Med.

[R13] Hamedi A, Lashgari AP, Pasdaran A (2019). Antimicrobial activity and analysis of the essential oils of
selected
endemic edible Apiaceae plants root from Caspian Hyrcanian region (North of
Iran). Pharm Sci.

[R14] Sertel S, Eichhorn T, Plinkert PK, Efferth T (2011). Chemical Composition and antiproliferative activity of essential
oil from
the leaves of a medicinal herb, Levisticum officinale, against UMSCC1 head
and neck
squamous carcinoma cells. Anticancer Res.

[R15] Shafaghat A (2011). Chemical constituents, antimicrobial and antioxidant activity of
the
hexane extract from root and seed of Levisticum persicum Freyn and Bornm. J Med Plants Res.

[R16] Moradalizadeh M, Akhgar MR, Rajaei P, Faghihi-Zarandi A (2012). Chemical composition of the essential oils of Levisticum
officinale
growing wild in Iran. Chem Nat Compd.

[R17] Khodashenas M, Keramat B, Emamipoor Y (2015). Callus induction and PLBs production from Levisticum officinale
koch (a
wild medicinal plant). J Appl Environ Biol Sci.

[R18] Spréa RM, Fernandes Â, Finimundy TC, et al (2020). Lovage (Levisticum officinale WDJ Koch) roots: A source of
bioactive
compounds towards a circular economy. Resources.

[R19] Miran M, Esfahani HM, Jung JH, et al (2020). Characterization and Antibacterial Activity of Phthalides from
the Roots
of the Medicinal Herb Levisticum officinale WDJ Koch. Iran J Pharm Res IJPR.

[R20] Mirjalili MH, Salehi P, Sonboli A, Hadian J, Ebrahimi SN, Yousefzadi M (2010). The composition and antibacterial activity of the essential oil
of
Levisticum officinale Koch flowers and fruits at different developmental
stages. J Serbian Chem Soc.

[R21] Aydin E, Türkez H, Geyikoğlu F (2013). Antioxidative, anticancer and genotoxic properties of α-pinene on
N2a
neuroblastoma cells. Biologia (Bratisl).

[R22] Bai X, Tang J (2020). Myrcene exhibits antitumor activity against lung cancer cells by
inducing
oxidative stress and apoptosis mechanisms. Nat Prod Commun.

[R23] Juliana de, de Carvalho, Daniele de, et al (2021). Mechanism of Action of Limonene in Tumor Cells: A Systematic
Review and
Meta-Analysis. Curr Pharm Des.

[R24] Złotek U, Lewicki S, Markiewicz A, Szymanowska U, Jakubczyk A (2021). Effects of Drying Methods on Antioxidant, Anti-Inflammatory, and
Anticancer Potentials of Phenolic Acids in Lovage Elicited by Jasmonic Acid
and
Yeast Extract. Antioxidants.

[R25] Liang N, Kitts DD (2015). Role of chlorogenic acids in controlling oxidative and
inflammatory
stress conditions. Nutrients.

[R26] Jiang Y, Nan H, Shi N, Hao W, Dong J, Chen H (2021). Chlorogenic acid inhibits proliferation in human hepatoma cells
by
suppressing noncanonical NF-κB signaling pathway and triggering
mitochondrial
apoptosis. Mol Biol Rep.

[R27] Feng R, Lu Y, Bowman LL, Qian Y, Castranova V, Ding M (2005). Inhibition of activator protein-1, NF-κB, and MAPKs and induction
of
phase 2 detoxifying enzyme activity by chlorogenic acid. J Biol Chem.

[R28] Abubakar AR, Haque M (2020). Preparation of medicinal plants: Basic extraction and
fractionation
procedures for experimental purposes. J Pharm Bioallied Sci.

[R29] Chomczynski P, Wilfinger W, Kennedy A, Rymaszewski M, Mackey K (2010). RNAzol® RT: a new single-step method for isolation of RNA. Nature Methods.

[R30] Kordestani Z, Shahrokhi-Farjah M, Yazdi Rouholamini, Saberi A (2020). Reduced ikk/nf-kb expression by Nigella sativa extract in breast
cancer. Middle East J Cancer.

[R31] Luo Y, Fu X, Ru R, et al (2020). CpG oligodeoxynucleotides induces apoptosis of human bladder
cancer cells
via caspase-3-Bax/Bcl-2-p53 axis. Arch Med Res.

[R32] Creagh EM, Conroy H, Martin SJ (2003). Caspase-activation pathways in apoptosis and immunity. Immunol Rev.

[R33] Amiri M, Nasrollahi F, Barghi S, et al (2018). The effect of ethanol baneh skin extract on the expressions of
bcl-2,
bax, and caspase-3 concentration in human prostate cancer pc3 cells. Int J Cancer Manag.

[R34] Gao C, Wang AY (2009). Significance of increased apoptosis and Bax expression in human
small
intestinal adenocarcinoma. J Histochem Cytochem.

[R35] Moretti A, Weig HJ, Ott T, et al (2002). Essential myosin light chain as a target for caspase-3 in failing
myocardium. Proc Natl Acad Sci.

[R36] Rajabi S, Maresca M, Yumashev AV, Choopani R, Hajimehdipoor H (2021). The most competent plant-derived natural products for targeting
apoptosis
in cancer therapy. Biomolecules.

[R37] Loizzo MR, Tundis R, Conforti F, et al (2010). Salvia leriifolia Benth (Lamiaceae) extract demonstrates in vitro
antioxidant properties and cholinesterase inhibitory activity. Nutr Res.

[R38] Bogucka-Kocka A, Smolarz HD, Kocki J (2008). Apoptotic activities of ethanol extracts from some Apiaceae on
human
leukaemia cell lines. Fitoterapia.

[R39] Sargazi S, Saravani R, Galavi H, Mollashahee-Kohkan F (2017). Effect of Levisticum officinale hydroalcoholic extract on DU-145
and PC-3
prostate cancer cell lines. Gene, Cell Tissue.

[R40] Jambor T, Arvay J, Tvrda E, Kovacik A, Greifova H, Lukac N (2021). The effect of Apium graveolens L, Levisticum officinale and
Calendula
officinalis L on cell viability, membrane integrity, steroidogenesis, and
intercellular communication in mice Leydig cells in vitro. Physiol Res.

[R41] Złotek U, Szymanowska U, Pecio Ł, Kozachok S, Jakubczyk A (2019). Antioxidative and potentially anti-inflammatory activity of
phenolics
from lovage leaves Levisticum officinale Koch elicited with jasmonic acid
and yeast
extract. Molecules.

[R42] Choi ES, Yoon JJ, Han BH, et al (2018). Ligustilide attenuates vascular inflammation and activates
Nrf2/HO-1
induction and, NO synthesis in HUVECs. Phytomedicine.

[R43] Chopra B, Dhingra AK, Dhar KL, Nepali K (2021). Emerging role of terpenoids for the treatment of cancer: A review. Mini Rev Med Chem.

[R44] Marques FM, Figueira MM, Schmitt EFP, et al (2019). In vitro anti-inflammatory activity of terpenes via suppression
of
superoxide and nitric oxide generation and the NF-κB signalling pathway. Inflammopharmacology.

[R45] Luo JL, Kamata H, Karin M (2005). IKK/NF-κB signaling: balancing life and death–a new approach to
cancer
therapy. J Clin Invest.

[R46] Harris RE (Published). Cyclooxygenase-2 (cox-2) and the inflammogenesis of cancer. Inflamm Pathog Chronic Dis COX-2 Controv.

[R47] Greenhough A, Smartt HJM, Moore AE, et al (2009). The COX-2/PGE 2 pathway: key roles in the hallmarks of cancer and
adaptation to the tumour microenvironment. Carcinogenesis.

[R48] Roelofs HMJ, Te Morsche, van Heumen, Nagengast FM, Peters WHM (2014). Over-expression of COX-2 mRNA in colorectal cancer. BMC Gastroenterol.

[R49] Conlon M (2011). Analysis of the phytochemical composition and anti-inflammatory
properties of Lovage. Published online.

[R50] Amraie E, Pouraboli I, Rajaei Z (2020). Neuroprotective effects of Levisticum officinale on LPS-induced
spatial
learning and memory impairments through neurotrophic, anti-inflammatory, and
antioxidant properties. Food Funct.

[R51] Sargazi ML, Saravani R, Shahraki A (2019). Hydroalcoholic extract of Levisticum officinale increases cGMP
signaling
pathway by down-regulating PDE5 expression and induction of apoptosis in
MCF-7 and
MDA-MB-468 breast cancer cell lines. Iran Biomed J.

